# Supplementary material for: Nf1 deficiency modulates the stromal environment in the pretumorigenic rat mammary gland
Source: Front Cell Dev Biol. 2024 May 10;12:1375441. doi: 10.3389/fcell.2024.1375441 (PMC11116614; doi:10.3389/fcell.2024.1375441)
Supplement: Supplementary file 1 [file DataSheet1.docx]

Supplementary Material

# Supplementary Figures

**Supplemental Figure 1. Aged IF/+ rats show have increased collagen with DCIS-like phenotype.** Representative images of H&E-stained tissue sections at 7 months old for WT animals and 7.5 months old for IF/+ rats. Scale bar = 100 µm.

**Supplemental Figure 2. Distinct collagen expression in different cell populations of the mammary gland.** Z-scores of collagen gene expression in tumors as well as adipocytes, epithelial cells, fibroblast, whole normal mammary gland before tumor formation at 33 days old.
